# Supplementary material for: Clinical and Morphological Features of ER-Positive HER2-Negative Breast Tumors with PIK3CA Mutations in Russian Patients
Source: Cancers (Basel). 2025 May 30;17(11):1833. doi: 10.3390/cancers17111833 (PMC12153798; doi:10.3390/cancers17111833)
Supplement: Supplementary file 1 [file cancers-17-01833-s001.zip › cancers-3655943-supplementary.pdf]

Supplementary material

Table S1. Description of methods for detection of *PIK3CA* mutations

Exons / Mutation	Primers	PCR composition	PCR conditions / Device
**High resolution melting (HRM) analysis**			
Exon 2 (codons 64-101)	Forward: [Biotin]CCCCTCCATCAACTTCTTCAA<br>Reverse: CGGTTGCCTACTGGTTCAAT	1 µl template, 0.15 u uracil-DNA glycosylase, 0.75 u hot-start polymerase, GeneAmp™ 10X PCR Buffer I (Applied Biosystems), 3.5 mM $MgCl_2$, 1x EvaGreen, 200 µM dNTPs, 0.3 µM primers in a total volume of 20 µl	DNA processing with uracil-DNA glycosylase: 15 min., 37°C; activation of Taq polymerase: 10 min., 95°C; then 50 cycles (denaturation: 15 s, 95°C; annealing: 30 s, 60°C; synthesis: 30 s, 72°C); then high-resolution melting analysis (temperature ramping from 65 to 95°C, rising by 0.07°C/1 s and fluorescence acquisition setting recommended by the manufacturer). Device: LightCycler® 96 Instrument (Roche)
Exon 2 (codons 98-117)	Forward: ACCTTCGGCTTTTTCAACC<br>Reverse: [Biotin]AGAAAGGGACAACAGTTAAGCT		
Exon 7_ (codons 418-441)	Forward: GACTAGTGAATATTTTTCTTTGTT<br>Reverse: [Biotin]ACTGGCCAAAGATTCAAAGC		
Exon 7_ (codons 425-455)	Forward: [Biotin]GGAACACTGTCCATTGGCATG<br>Reverse: CCAGTAACACCAATAGGGTTCAG		
Exon 10 (codons 526-552)	Forward: CTAGAGACAATGAATTAAGGGAA<br>Reverse: [Biotin]CATTTTAGCACTTACCTGTGAC		
Exon 20 (codons 1027-1057)	Forward: TTGCATACATTCGAAAGACCC<br>Reverse: [Biotin]TGCTGTTTAATTGTGTGGAAGA		
**Allelic discrimination by TaqMan-PCR**			

N345K	Forward: ATAAATAGTGCACTCAGAATAA<br>Reverse: ATCAGCATTTGACTTTACC<br>Probes:<br>N345N: [FAM]CCTACGTGAATGTAAATATTCG[BHQ1]<br>N345K: [JOE]CCTACGTGAAAGTAAATATTCG[BHQ1]	1 µl template, 0.75 u hot-start polymerase, GeneAmp™ 10X PCR Buffer I (Applied Biosystems), 3.5 mM $MgCl_2$, 200 µM dNTPs, 0.3 µM primers and probes in a total volume of 20 µl	Activation of Taq polymerase: 10 min., 95°C; then 50 cycles (denaturation: 15 s, 95°C; annealing/synthesis: 60 s, 58°C).<br>Device: CFX-96 (Bio-Rad)
E726K	Forward: CATTAACTTAACTGACATTCTC<br>Reverse: GAAAAGAGTCTCAAACACAA<br>Probes:<br>E726E: [FAM]AGAAGAAGGATGAAACACAAAAG[BHQ1]<br>E726K: [JOE]AGAAGAAGGATAAAACACAAAAG[BHQ1]		Activation of Taq polymerase: 10 min., 95°C; then 50 cycles (denaturation: 15 s, 95°C; annealing/synthesis: 60 s, 59°C).<br>Device: CFX-96 (Bio-Rad)
G118D	Forward: ATGCTGTGTATGTAATAGAATGTT<br>Reverse: TTAACCATATCAAATTCACACACT<br>Probes:<br>G118G: [FAM]ATTAAAGGTTTTGCTATCGGCAT[BHQ1]<br>G118D: [HEX]ATTAAAGATTTTGCTATCGGCATGC[BHQ1]		Activation of Taq polymerase: 10 min., 95°C; then 50 cycles (denaturation: 15 s, 95°C; annealing/synthesis: 60 s, 62°C).<br>Device: CFX-96 (Bio-Rad)
E453K/Q	Forward: GGAACACTGTCCATTGGCATG<br>Reverse: CCAGTAACACCAATAGGGTTCAG<br>Probes:<br>PIK3CA_E453E: [FAM]CCAGTACCTCATGGATTAGAAGA[BHQ1]<br>PIK3CA_E453K: [JOE]CCAGTACCTCATGGATTAAAAGA[BHQ1]<br>PIK3CA_E453Q: [CY35]CCAGTACCTCATGGATTACAAGA[BHQ2]		Activation of Taq polymerase: 10 min., 95°C; then 50 cycles (denaturation: 15 s, 95°C; annealing/synthesis: 60 s, 61°C).<br>Device: CFX-96 (Bio-Rad)
C420R	Forward: GACTAGTGAATATTTTTCTTTGTT<br>Reverse: ACTGGCCAAAGATTCAAAGC<br>Probes:<br>PIK3CA_C420C: [FAM]AGGAACACTGTCCATTGGCA[BHQ1]<br>PIK3CA_C420R: [HEX]AGGAACACCGTCCATTGGC[BHQ1]		Activation of Taq polymerase: 10 min., 95°C; then 50 cycles (denaturation: 15 s, 95°C; annealing/synthesis: 60 s, 61°C).<br>Device: CFX-96 (Bio-Rad)

R108-K111	Forward: ACCTTCGGCTTTTTCAACC<br>Reverse: AGAAAGGGACAACAGTTAAGCT<br>Probes:<br>PIK3CA_R108R: [FAM]AGTAGGCAACCGTGAAGAAAAGAT[BHQ1]<br>PIK3CA_R108H: [HEX]AGTAGGCAACCATGAAGAAAAGAT[BHQ1]<br>PIK3CA_K111del: [CY5.5]AGTAGGCAACCGTGAAGAAATC[BHQ3]<br>PIK3CA_E110del: [ROX]AGTAGGCAACCGTGAAAAGATC[BHQ2]<br>PIK3CA_K111E: [CY5]ACCGTGAAGAAGAGATCCTCAATC[BHQ2]	Activation of Taq polymerase: 10 min., 95°C; then 50 cycles (denaturation: 15 s, 95°C; annealing/synthesis: 60 s, 63°C).<br>Device: CFX-96 (Bio-Rad)
E81K, R88Q	Forward: CCCCTCCATCAACTTCTTCAA<br>Reverse: CGGTTGCCTACTGGTTCAAT<br>Probes:<br>PIK3CA_R88R: [FAM]GAAACAAGACGACTTTGTGACCTT[BHQ1]<br>PIK3CA_R88Q: [HEX]TGAAACAAGACAACTTTGTGACCTT[BHQ1]<br>PIK3CA_E81E: [FAM]GCAGAAAGGGAAGAATTTTTTGATGA[BHQ1]<br>PIK3CA_E81K: [HEX]AGCAGAAAGGGAAAAATTTTTTGATGA[BHQ1]	Activation of Taq polymerase: 10 min., 95°C; then 50 cycles (denaturation: 15 s, 95°C; annealing/synthesis: 60 s, 63°C).<br>Device: CFX-96 (Bio-Rad)
E542/E545/Q546	Forward: CTAGAGACAATGAATTAAGGGAA<br>Reverse: CATTTTAGCACTTACCTGTGAC<br>Probes:<br>PIK3CA_E542E: [FAM]TCCTCTCTCTGAAATCACTGAGC[BHQ1]<br>PIK3CA_E542K: [HEX]TCCTCTCTCTAAAATCACTGAGCA[BHQ1]<br>PIK3CA_E545E: [FAM]CTGAAATCACTGAGCAGGAGAAAG[BHQ1]<br>PIK3CA_E545A: [ROX]AAATCACTGCGCAGGAGAAAg[BHQ2]<br>PIK3CA_E545K: [HEX]CTGAAATCACTAAGCAGGAGAAAGA[BHQ1]<br>PIK3CA_E545G: [CY5]TGAAATCACTGGGCAGGAGAAA[BHQ2]<br>PIK3CA_E545D: [CY5.5]TCTGAAATCACTGACCAGGAGAAAG[BHQ3]<br>PIK3CA_Q546Q: [FAM]AAATCACTGAGCAGGAGAAAGATTT[BHQ1]<br>PIK3CA_Q546K: [HEX]TGAAATCACTGAGAAGGAGAAAGA[BHQ1]<br>PIK3CA_Q546P: [CY5]AAATCACTGAGCCGGAGAAAGAT[BHQ2]<br>PIK3CA_Q546R: [ROX]AAATCACTGAGCGGGAGAAAGAT[BHQ2]<br>PIK3CA_Q546E: [CY5.5]GAAATCACTGAGGAGGAGAAAGATTT[BHQ3]	Activation of Taq polymerase: 10 min., 95°C; then 50 cycles (denaturation: 15 s, 95°C; annealing/synthesis: 60 s, 61°C).<br>Device: CFX-96 (Bio-Rad)
M1043/N1044/H1047/G1049	Forward: TTGCATACATTCGAAAGACCC<br>Reverse: TGCTGTTTAATTGTGTGGAAGA<br>Probes:<br>PIK3CA_H1047H: [FAM]ATGCACATCATGGTGGCT[BHQ1]<br>PIK3CA_H1047R: [R6G]ATGCACGTCATGGTGGCT[BHQ1]<br>PIK3CA_H1047L: [ROX]ATGCACTTCATGGTGGCT[BHQ1]<br>PIK3CA_M1043M: [FAM]TCATGAAACAAATGAATGATGCACA[BHQ1]<br>PIK3CA_M1043It: [R6G]TCATGAAACAAATTAATGATGCACATC[BHQ1]<br>PIK3CA_M1043Ia:[R6G]TCATGAAACAAATAAATGATGCACATC[BHQ1]	Activation of Taq polymerase: 10 min., 95°C; then 50 cycles (denaturation: 15 s, 95°C; annealing/synthesis: 60 s, 61°C).<br>Device: CFX-96 (Bio-Rad)

	PIK3CA_M1043Ic:[R6G]TCATGAAACAAATCAATGATGCACA[BHQ1]<br>PIK3CA_M1043V:[ROX]TCATGAAACAAGTGAATGATGCACA[BHQ2]<br>PIK3CA_N1044K: [CY5]TCATGAAACAAATGAAAGATGCACA[BHQ2]<br>PIK3CA_N1044Y: [CY5.5]TCATGAAACAAATGTATGATGCACA[BHQ3]<br>PIK3CA_G1049G: [FAM]GCACATCATGGTGGCTGG[BHQ1]<br>PIK3CA_G1049R: [R6G]GCACATCATCGTGGCTGG[BHQ1]		
**Digital droplet PCR**			
E542K, E545K	Forward: CTAGAGACAATGAATTAAGGG<br>Reverse: CATTTTAGCACTTACCTGTGAC<br>Probes:<br>E542E [FAM]TCCTCTCTCTGAAATCACTGAG[BHQ1]<br>E545E [FAM]AAATCACTGAGCAGGAGAAAGA[BHQ1]<br>E542K [JOE]TCCTCTCTCTAAAATCACTGAGC[BHQ1]<br>E545K [JOE]TGAAATCACTAAGCAGGAGAAAGA[BHQ1]	2 µl template, 4 µl ddPCR Supermix for Probes (no dUTP) (Bio-Rad), 0.5 µM primers and probes in a total volume of 20 µl	Activation of Taq polymerase: 10 min., 95°C; then 50 cycles (denaturation: 30 s, 94°C; annealing/synthesis: 60 s, 56°C), then heating: 10 min., 98°C.<br>Device: T100 Thermal Cycler (Bio-Rad); QX200 Droplet Digital PCR System (Bio-Rad)
H1047R, H1047L	Forward: GCATACATTCGAAAGACC<br>Reverse: GCTGTTTAATTGTGTGGAA<br>Probes:<br>H1047H [FAM]GATGCACATCATGGTGGC[BHQ1]<br>H1047R [JOE]GATGCACGTCATGGTGGC[BHQ1]<br>H1047L [JOE]TGATGCACTTCATGGTGGC[BHQ1]		

Table S2. List of double mutations in the *PIK3CA* gene

Mutation	Number of cases
E545K+E726K	6
E726K+H1047R	6
E542K+E726K	4
R108H+H1047R	2
C420R+H1047R	2
E453K+E542K	2
E453K+H1047R	2
E542K+H1047R	2
E545K+H1047R	2
C420R+E726K	1
C420R+G1049R	1
E81K+H1047L	1
D84H+H1047L	1
K111del+H1047R	1
G118D+E453K	1
G118D+E542K	1
G118D+H1047R	1
N345K+E545K	1
N345K+Q546K	1
E418K+H1047R	1
L422W+H1047Y	1
E453Q+E545K	1
E453Q+H1047R	1
E545Q+H1047R	1
E726K+H1047L	1
M1043I+N1044H	1
M1043V+N1044Y	1
